# Out-of-pocket expenditures on over-the-counter medications: a cross-sectional study of consumer spending patterns

**DOI:** 10.3389/fpubh.2026.1777770

**Published:** 2026-03-27

**Authors:** Hadel Fuad Alzuabi, Vineetha Bharathan Menon, Jisha Myalil Lucca

**Affiliations:** 1 Department of Pharmaceutical Sciences, College of Pharmacy, Gulf Medical University, Ajman, United Arab Emirates; 2 Department of Pharmacy Practice, College of Pharmacy, Gulf Medical University, Ajman, United Arab Emirates

**Keywords:** consumer satisfaction, out-of-pocket expenditure, over-the-counter medications, self-medication, United Arab Emirates

## Abstract

**Background:**

Over-the-counter (OTC) medications are widely used, yet little is known about their cost implications and consumer satisfaction in the United Arab Emirates (UAE). This study assessed self-medication practices, out-of-pocket (OOP) expenditure, and satisfaction among the general population and patients with cardiovascular diseases.

**Methods:**

A cross-sectional survey was conducted using a structured questionnaire among adult residents of the UAE (≥18 years) who had used OTC. A total of 206 participants, from the general public, were recruited. Exclusion criteria included Participants without internet and individuals with incomplete responses. Data on demographic characteristics, OTC and herbal medication use, 30-day OOP expenditure, and satisfaction with costs were collected. Descriptive statistics and regression analyses were performed.

**Results:**

Among 206 participants, who reported regular use of over-the-counter (OTC) medications. Usage was more frequent among females (*n* = 101, 49%), and higher among employed individuals (*n* = 128, 62%). Analgesics, antipyretics, and respiratory treatments were the most commonly used OTC products. Overall satisfaction with OTC medications was high. The overall total OOP expenditure was highest in the 100–499 AED (27–135 USD) range (54%). For acute illnesses, the 50–99 AED (14–26 USD) range was most common (28.64%). Satisfaction with OTC medication costs is shaped by a complex interaction of demographic, socioeconomic, and clinical factors.

**Conclusion:**

Self-medication with OTC products is common among adults in the UAE, particularly among females. While overall satisfaction with OTC medication costs is high, a substantial proportion of participants incur moderate out-of-pocket expenses. These findings highlight the need for public awareness on cost-effective self-medication practices and further research on financial implications of OTC use.

## Introduction

Medications are the most significant contributors to healthcare costs, especially when out-of-pocket (OOP) payments are considered (1, 2). Globally, the over-the-counter (OTC) drug market is substantial and rapidly expanding. In 2025, the OTC market size was estimated at USD 135.25 billion and is projected to reach approximately USD 242.94 billion by 2034, growing at a compound annual growth rate (CAGR) of 6.72% (3). The growth of the OTC medication market is influenced by several factors, including increased self-medication practices, prescription-to-OTC switches, and rising health literacy through online resources and consumer behaviors (4–6). Regulatory reforms, changes in dispensing and distribution systems, and retail market dynamics have also affected OTC availability and pharmacy performance in many settings (7, 8). Decision factors that influence OTC selection—especially among older adults—and patterns of OTC continuation versus prescribed therapy further shape consumer willingness to pay for these products despite potential cumulative financial impacts over time (9, 10).

Evidence from regional cross-sectional studies indicates that community pharmacies are the primary source of OTC medications for most consumers. For instance, research conducted in Saudi Arabia reported that OTC and herbal medications were predominantly purchased directly from community pharmacies (6, 11).

In several countries, the financial burden of medications is particularly evident. For example, studies of multimorbidity and household medicine spending highlight substantial OOP burdens for families with older members (1). Similarly, analyses of healthcare expenditures associated with chronic conditions such as cardiovascular disease underscore sizable hospital costs that are often compounded by additional, less-tracked OOP spending on OTC treatments and supplements (1). In parts of the world, spending on complementary, herbal, and natural products is substantial and contributes meaningfully to household OOP expenditures; population studies from Saudi Arabia and Jordan document high prevalence and expenditure on herbal and complementary medicines (11, 12). High direct medication costs have been associated with reduced adherence to prescribed therapies in several settings, particularly among patients with limited financial means (2, 10).

In the Middle East there has been a noticeable surge in the use of OTC and herbal products; market analyses and regional studies report rising market value and widespread consumer use of herbal products in nearby countries (3, 11, 12). Despite this growth, the majority of OTC purchases in many countries continue to be made through direct out-of-pocket payments as OTC and herbal products are typically not covered by insurance or public reimbursement programs and regulatory frameworks vary between jurisdictions (1, 2, 7).

Out-of-pocket spending on OTC medications remains an underexplored but increasingly relevant dimension of healthcare costs—especially for chronic disease management and routine self-care. For patients with chronic conditions, these expenses can accumulate significantly over time, particularly when multiple OTC products are used concurrently (1, 2). At the same time, individuals from the general population also spend considerably on OTC medications, but their purchasing behaviors, motivations, and patterns of use may differ from those with chronic illnesses (6, 11, 12). As healthcare systems increasingly promote self-care and responsible medication use, it is crucial to assess whether financial barriers limit access to necessary OTC products or encourage inappropriate self-medication. This study therefore aims to quantify the magnitude and patterns of out-of-pocket OTC medication expenditures, identify demographic and behavioral factors influencing spending, determine the types of products most frequently purchased, and evaluate participant satisfaction with the amounts they spend.

## Methodology

### Study design

A cross-sectional study was conducted to assess the magnitude and patterns of out-of-pocket (OOP) expenditures on over-the-counter (OTC) medications.

### Study setting and duration

Data were collected over a six-month period, from January 2025 to June 2025, across multiple community-based settings in the United Arab Emirates (UAE), including community pharmacies, outpatient clinics, and wellness centres, to ensure a diverse and representative sample.

### Study population and sampling

Participants included in this study were adults aged 18 years and above who had purchased at least one over-the-counter (OTC) medication in the past month and provided informed consent to participate. Individuals were excluded if they were unable to recall their recent OTC purchases or if they were healthcare professionals or pharmacy staff, to minimize potential professional bias in reporting behavior or expenditure.

### Sample size

The sample size was calculated *a priori* based on estimation of a population proportion. A 95% confidence level and a margin of error of 5% were assumed. In the absence of precise prior estimates for the primary outcomes, a conservative expected prevalence of 50% was used to maximize the required sample size. An effectively unlimited population size (>20,000) was assumed. Based on these assumptions, the minimum required sample size was 384 participants.

### Variables and operational definitions

The primary outcome variable of this study was the total out-of-pocket (OOP) expenditure on over-the-counter (OTC) medications during the preceding 30 days, measured in United Arab Emirates Dirhams (AED) and analyzed as a continuous variable. Secondary outcome variables included the types of OTC products purchased, which were categorized according to their therapeutic class, the purpose for OTC use, and participants’ satisfaction with the costs of OTC medications. Satisfaction was assessed using a 5-point Likert scale ranging from “very dissatisfied” to “very satisfied.”

### Data collection tool

A structured questionnaire was developed specifically for this study and was pre-tested on a small group of respondents to ensure clarity, relevance, and ease of understanding. The first section collected sociodemographic information, including age, gender, income, education level, and employment status. The second section focused on participants’ health profiles, such as the presence of chronic medical conditions. The third section gathered detailed information on OTC medication use, including out-of-pocket expenditures over the past 30 days. The fourth section examined the types of products purchased and the reasons for their use. Finally, the last section assessed participants’ satisfaction with the costs of OTC medications.

Data were collected through face-to-face interviews conducted by trained researchers in community-based settings. Additionally, a digital version of the questionnaire was made available to accommodate broader participation. In cases where the digital format was used, follow-up validation was conducted via phone or email to ensure data accuracy and completeness. To minimize recall bias, the reporting period was limited to 30 days.

### Data analysis

Data were entered into SPSS version 28.0 for analysis. Descriptive statistics (means, medians, and frequencies) summarized OOP expenditure and product types. Chi-square tests and *t*-tests were used to examine associations between spending and demographic variables. A multivariate linear regression model identified significant predictors of higher OOP spending.

### Ethical considerations

Ethical approval was obtained from the institutional review board (IRB) at Gulf Medical University with a Ref. no. “IRB-COP-STD-34-Sept-2024.” Participation was voluntary, and informed consent was obtained from all respondents. Data confidentiality and anonymity were strictly maintained throughout the study.

## Results

A total of 327 individuals were screened for the study. Of these, 121 were excluded—120 due to non-use of over-the-counter (OTC) medications and 1 who declined participation. A total of 206 participants were included in the final analysis of OOP expenditure and product types.

### Study population and sociodemographic characteristics

The study sample (*n* = 206) was demographically diverse, with an almost equal gender distribution. Most participants were employed [*n* = 128, (62%)] and lived in medium-sized households of 4–8 members [*n* = 131, (63.59%)]. Income levels varied widely, with a total of [*n* = 71, (34.47%)] reporting moderate earnings more than 10,000 AED (≈ USD 2,700). Participants commonly reported taking multiple medications during illness, with the majority [*n* = 146, (70.87%)] using 2–4 medications daily. Participant sociodemographic details are given in Table 1.

**Table 1:** Participants’ sociodemographic and clinical characteristics.

Characteristics	Total, (*N* = 206)
Gender	
Female	101 (49.03)
Male	105 (50.97)
Race/ethnicity	
Middle East	166 (80.58)
South Asia	29 (14.08)
Southeast Asia	7 (3.40)
Others	4 (1.94)
Employment status	
Employed	128 (62.14)
Unemployed	21 (10.19)
Retired	41 (19.90)
Student	16 (7.77)
Monthly income	
Less than AED 5000	24 (11.65)
AED 5000–10,000	52 (25.24)
More than AED 10000	71 (34.47)
No income currently	40 (19.42)
Prefer not to mention	19 (9.22)
Number of family members per household	
1 member	11 (5.34)
2–3 members	48 (23.30)
4–8 members	131 (63.59)
More than 8 members	16 (7.77)
Number of medications taken by participants	
1	34 (16.50)
2–4	146 (70.87)
5 or more	26 (12.62)

### Patterns of OTC medication use and out-of-pocket expenditure

**Figure 1:**
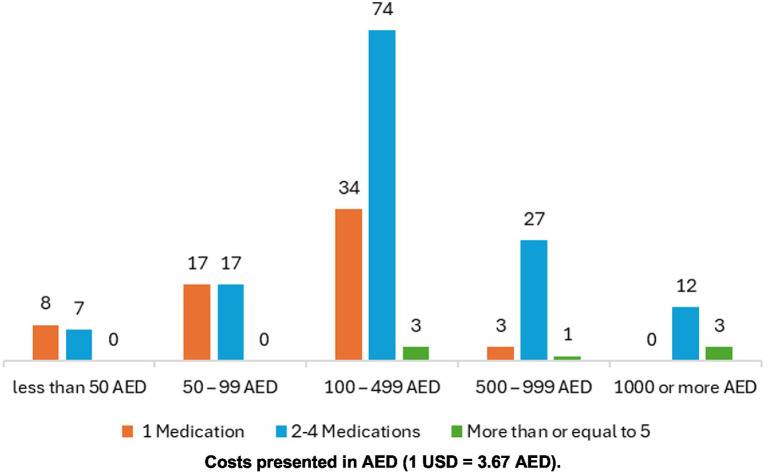
Out-of-pocket (OOP) expenditure vs. number of OTC medications.

Most participants reported using 2–4 OTC medications, with utmost expenditures concentrated in the AED 100–499 range. Among participants using a single OTC medication (*n* = 62), more than half [*n* = 34 (54.8%)] reported moderate spending of AED 100–499. A similar pattern was observed in the 2–4 medication group, where the largest proportion [*n* = 74 (54%)] also spent AED 100–499. Overall, only a small proportion of participants [*n* = 15 (7%)] incurred high costs of AED 1,000 or more. Detailed information on participants’ OTC spending behavior is presented in Figure 1.

#### Monthly out-of-pocket expenditure by medication category

The top used OTC categories were antitussives (*n* = 196, 95.15%); multivitamin and micronutrient supplements (*n* = 193, 93.69%); and antipyretics and analgesics medications (*n* = 184, 89.32%). Within these categories, over half of participants reported spending in the AED 100–499 range. [*n* = 107 (51.94%), *n* = 106 (51.45%), and *n* = 96 (46.60%)] respectively indicating a dominant middle-range financial burden. Less frequently used product, such as aphrodisiac formulations (*n* = 43, 20.87%) and sedative-hypnotics (*n* = 92, 44.66%), also showed a concentration of users [*n* = 25 (25.24%) and *n* = 50 (24.27%), respectively] in the AED 100–499 range, suggesting that even among smaller user groups, moderate expenditure was common. For drugs targeting gastrointestinal disorders (*n* = 173, 83.98%) and phytopharmaceutical/botanical extracts (*n* = 186, 90.29%), the majority similarly clustered in the AED 100–499 bracket [*n* = 94 (45.63%) and *n* = 99 (48.06%), respectively]. Notably, a consistent minority [*n* = 61(4)] across several classes reported spending ≥1,000 AED monthly, highlighting a small but significant segment of high-expenditure consumers. These patterns are summarized in Table 2.

**Table 2:** Monthly out-of-pocket expenditure vs. OTC medications categories.

Monthly Spending range (AED)^†^	Antipyretics, analgesics, and anti-inflammatory drugs^a^, *n* (%)	Antitussives, expectorants, and decongestants^b^, *n* (%)	Multivitamin and micronutrient supplements^c^, *n* (%)	Aphrodisiac formulations^d^, *n* (%)	Drugs for gastrointestinal disorders n^e^, (%)	Phytopharmaceuticals and botanical extracts^f^, *n* (%)	Sedative-hypnotics and anxiolytic agents^g^, *n* (%)
<50 AED	14 (6.79)	14 (6.79)	12 (5.83)	2 (0.97)	12 (5.83)	12 (5.83)	4 (1.94)
50–99 AED	30 (14.56)	30 (14.56)	31 (15.05)	3 (1.45)	29 (14.29)	31 (15.05)	13 (6.31)
100–499 AED	96 (46.60)	107 (51.94)	106 (51.45)	25 (25.24)	94 (45.63)	99 (48.06)	50 (24.27)
500–999 AED	30 (14.56)	31 (15.05)	30 (15.54)	7 (3.39)	28 (13.59)	30 (14.56)	15 (7.28)
≥1,000 AED	14 (6.79)	14 (6.79)	14 (6.79)	6(2.91)	10 (4.85)	14 (6.79)	10 (4.85)
Total number of participants *N* (%)	*N* = 184 (89.32)	*N* = 196 (95.15)	*N* = 193 (93.69)	*N* = 43 (20.87)	*N* = 173 (83.98)	*N* = 186 (90.29)	*N* = 92 (44.66)

^†^Participants selected multiple medication classes.

a Antipyretics/analgesics: Paracetamol, Ibuprofen, Aspirin, Naproxen, Ketoprofen.

b Antitussives/expectorants/decongestants: Dextromethorphan, Guaifenesin, Ambroxol, Phenylephrine, Pseudoephedrine.

c Multivitamins: multivitamin complexes, vitamin C, vitamin D, Calcium+D, Omega-3.

d Aphrodisiac formulations: L-arginine, *Panax ginseng*, Maca root, Yohimbe, Tribulus.

e GI disorders: Antacids, Loperamide, Bismuth subsalicylate, Probiotics, ORS.

f Phytopharmaceuticals: Echinacea, Ginger, Peppermint oil, Turmeric, *Ginkgo biloba*.

g Sedative-hypnotics/anxiolytic: Diphenhydramine, Doxylamine, Valerian, Melatonin, Chamomile.

### Expenditure on OTC based on purpose of use

Monthly out-of-pocket expenditure varied according to the purpose of OTC medication use. For chronic conditions, a substantial proportion [*n* = 84 (40.78%)] of participants reported no monthly spending. Spending for acute illnesses was more common, with most participants clustering in the lower-to-middle expenditure range of AED 1–499. Preventive and wellness products showed a bimodal distribution, with 50–99 AED and 100–499 AED being the most frequent ranges (each *n* = 70, 33.98%) When considering total out-of-pocket spending across all purposes, the AED 100–499 range was the most frequently reported category, accounting for over half of participants. [*n* = 111(53.88%)]. Details are given Table 3.

**Table 3:** Monthly out-of-pocket expenditure vs. purpose of OTC medication use.

Monthly spending range (AED)	Chronic diseases, *N* (%)	Acute illnesses, *N* (%)	Preventive and wellness products, *N* (%)	Total, *N* (%)
0	84 (40.78)	51 (24.76)	25 (12.14)	0 (0)
1–49	23 (11.17)	42 (20.39)	29 (14.08)	15 (7.28)
50–99	34 (16.50)	59 (28.64)	70 (33.98)	34 (16.50)
100–499	46 (22.33)	50 (24.27)	70 (33.98)	111 (53.88)
500–999	17 (8.25)	3 (1.46)	11 (5.34)	31 (15.05)
≥1,000	2 (0.97)	1 (0.49)	1 (0.49)	15 (7.28)
Most common range^a^	0	50–99	50–99 and 100–499	100–499
Proportion with any spending (%)	59.22	75.24	87.86	

Costs presented in AED (1 USD = 3.67 AED).

a The “Most common range” refers to the expenditure bracket with the highest number of respondents in each category. In cases where two ranges had the same highest frequency, both are reported.

### Factors associated with satisfaction regarding OTC costs

Gender was significantly associated with satisfaction (*p* = 0.0242), with a higher proportion of males reporting dissatisfaction compared to females. Race or ethnicity showed a strong association (*p* = 0.0001), as Middle Eastern participants were more likely to be dissatisfied than other groups. Employment status was also highly significant (*p* < 0.0001), with employed respondents reporting more dissatisfaction, while students tended to be more satisfied. Monthly income was significantly linked to satisfaction (*p* < 0.0001); individuals earning more than AED 10,000 reported higher satisfaction, whereas those without current income expressed greater dissatisfaction. Household size demonstrated a significant relationship (*p* = 0.0349), with larger households (4–8 members) showing a higher frequency of dissatisfaction. The number of medications taken daily (*p* = 0.001) and the number of OTC medications used (*p* = 0.0007) were both significantly related to dissatisfaction, especially among those taking 2–4 medications. Medical insurance coverage for OTC medications was a major determinant of satisfaction (*p* < 0.0001); participants with insurance coverage were more satisfied, while those without coverage or who were unsure reported higher dissatisfaction. Monthly out-of-pocket costs had a strong association with dissatisfaction (*p* < 0.0001), with higher spending corresponding to less satisfaction. Finally, although the perceived occurrence of adverse drug reactions due to OTC medications did not reach statistical significance (*p* = 0.0585), there was a trend toward increased dissatisfaction among those reporting ADRs. Associations between participant characteristics and satisfaction with OTC medication costs is given in Table 4.

**Table 4:** Factors affecting the customer satisfaction with cost of OTC Products.

Variables	Customer satisfaction			*p-*value
	Satisfied	Neutral	Unsatisfied	
Gender^a^				
Male	27	11	67	0.0242*
Female	24	25	52	
Race/ethnicity^b^				
Middle East	47	23	96	0.0001*
South Asian	3	10	16	
Southeast Asian	0	1	6	
Others	1	2	1	
Employment status^b^				
Employed	29	21	96	0.0000*
Unemployed	4	5	16	
Retired	11	4	6	
Student	7	6	1	
Monthly income^b^				
Less than AED 5000	5	9	10	0.000*
AED 5000–10,000	12	8	32	
More than AED 10000	22	11	38	
No income currently	5	3	32	
Prefer not to mention	7	5	7	
Number of family members per household^b^				
1 member	3	0	8	0.0349*
2–3 members	12	9	27	
4–8 members	31	26	74	
More than 8 members	5	1	10	
Number of medications taken by participants^b^				
1	10	11	13	0.0010*
2–4	33	24	89	
5 or more	8	1	17	
Number of OTC medications taken by participants^b^				
1	21	15	26	0.0007*
2–4	27	21	89	
5 or more	3	0	4	
Self-reported medical insurance coverage for OTC medications^b^				
Insurance covers OTC	30	10	3	0.0000*
Insurance does not cover OTC	13	16	98	
Do not know if insurance covers OTC	4	4	2	
No insurance	4	6	16	
Monthly out-of-pocket cost for OTC/herbal medications^b^				
Less than 50	8	3	4	0.0000*
50–99	18	6	10	
100–499	19	23	69	
500–999	3	3	25	
1,000 or more	3	1	11	
Perceived occurrence of ADRs due to OTC medications^b^				
Yes	11	4	31	**0.0585**
No	40	32	88	

ADRs, adverse drug reactions; OTC, over-the-counter.

a Chi square test.

b Fisher exact test.

Bold values represent *p*-values.*indicates statistical significance at *p* < 0.05.

## Discussion

This study aimed to explore the magnitude and patterns of out-of-pocket (OOP) expenditures on over-the-counter (OTC) medications, identify demographic factors influencing spending behavior, and determine the types of products most frequently purchased, as well as participants’ satisfaction with the amounts spent. By examining these aspects in a community-based sample, the study provides insight into consumer self-medication practices, economic burden, and preferences in OTC medication use. Understanding these patterns is essential for informing public health strategies, guiding pharmacists’ counselling, and optimizing safe and cost-effective use of OTC products.

Compared with national UAE demographics, our sample shows several notable differences. While the UAE population is male-dominant (≈64% male) our sample was nearly balanced (13). Several global studies, also report a higher prevalence of female participants in self-medication or OTC use, this discrepancy may be attributed to differences in the study populations, including cultural factors, access the healthcare, and gender-specific health-seeking behaviors. For example, a study conducted by Orayj et al. (6) in Saudi Arabia reported that females were more likely to engage in self-medication practices, driven by perceived autonomy in managing minor illnesses. Similarly, one study in Nigeria reported that self-medication prevalence was highest among females by more than half of their entire population (14). Ethnically, our participants were predominantly Middle Eastern (80.6%), whereas national data indicate that expatriates comprise about 88.5% of the UAE population, with South Asians forming the largest groups; thus, Middle Eastern residents were over-represented and South Asians under-represented in our study (13).

Employment status was a key determinant in our study, with 62% of participants employed. Several other previous studies have demonstrated that employment and economic stability are strongly associated with both the ability and willingness to purchase OTC medications (9). Our results further indicate that participants with moderate higher income—35% earning more than AED 10,000 per month—may have greater capacity to acquire OTC products, potentially contributing to higher out-of-pocket expenditures (1, 2). However, the notable proportion of participants with low or no income (27%) underscores that OTC use is not limited to higher-income groups. This pattern aligns with prior studies reporting widespread self-medication practices across diverse socioeconomic strata (3, 12), suggesting that cultural factors, perceived need, and accessibility may also play significant roles in OTC utilization.

Household size may also influence patterns of OTC medication use. In our study, the majority of participants (64%) lived in households with 4–8 members, while 23% resided in smaller households of 2–3 members. Larger households may facilitate the sharing of health knowledge and experiences related to common OTC medications, potentially increasing both awareness and use of these products (11). Additionally, larger households may experience more frequent minor illnesses among members, which could further contribute to higher OTC consumption.

The relationship between the number of OTC medications used and the corresponding expenditure patterns provides meaningful insight into consumer behavior. One Half of the participants using a single OTC medication reported moderate spending (100–499 AED), with no cases of extremely high expenditure (≥1,000 AED). This suggests that single-product use is generally associated with controlled costs and minimal economic impact, likely reflecting purchases for acute or self-limiting conditions. A similar pattern of spending is observed in other studies conducted in community settings (1, 2, 9). In contrast, participants using 2–4 OTC medications demonstrated greater variability in spending, with some reporting expenditures of ≥1,000 AED. This pattern indicates that while moderate spending remains dominant in this group, the likelihood of higher out-of-pocket costs increases as the number of products used rises. This may reflect more complex health needs, broader reliance on self-medication for symptom management, or the purchase of premium-priced health and wellness products. The small group of participants using five or more OTC medications showed the most striking distribution, with nearly 38% reporting very high expenditures (≥1,000 AED). This suggests a clear association between polypharmacy in OTC use and substantial financial burden. Such findings raise concerns about potential overuse, unnecessary duplication of therapies, and risks of drug–drug interactions.

As seen in other literature, the most frequently utilized OTC categories in this study were antitussives and decongestants; multivitamin and micronutrient supplements; and antipyretics/analgesics/anti-inflammatory agents, which were reported by nearly all participants. This reflects their widespread role in self-care for common conditions such as respiratory illnesses, nutritional supplementation, and pain or fever management. Importantly, in each of these categories, nearly one half of the respondents reported expenditures in the 100–499 AED range, suggesting that these commonly used products contribute most significantly to the overall financial burden of OTC medication use. This contrasts with the study conducted by Neufingerl and Eilander (15), where gastrointestinal medications represented the highest expenditure category, followed by pain relievers.

Less frequently used categories, including herbal products from aphrodisiac formulations such as ginkgo biloba and black cohosh, and sedative-hypnotics such as valerian and doxylamine, showed similar spending trends, with more than half of users in these groups also clustered in the 100–499 AED range. This indicates that even for medications with lower prevalence of use, the associated costs remain substantial for individual consumers. Such findings may point to high unit costs for certain products or regular use among a smaller but more financially invested subset of participants.

The analysis of OOP expenditure across chronic conditions, acute illnesses, and preventive/wellness products reveals distinct spending patterns that provide insight into consumer behavior. For chronic health conditions, 40% of the participants are not spending on OTC use, suggesting that individuals with chronic illnesses may primarily rely on prescribed medications covered by insurance or provided through healthcare facilities, with minimal dependence on OTC options. This aligns with findings from prior studies indicating that prescription medications dominate chronic disease management, while OTC medications play a supplementary role (1, 2).

In contrast, approximately three-fourths of participants who reported using OTC medications for acute illnesses indicated that their expenditure most commonly fell within the 1–499 AED range. This likely reflects the episodic yet frequent use of short-term symptomatic treatments such as antipyretics, analgesics, and cough and cold preparations. Acute illnesses are generally self-limiting in nature; therefore, consumers tend to purchase OTC medications for brief durations rather than prolonged use. The moderate level of expenditure observed in this group may indicate both the affordability and widespread accessibility of OTC remedies, which facilitate prompt self-management without the need for physician consultation. These findings are consistent with previous literature suggesting that OTC utilization for minor acute conditions is driven by convenience, cost-effectiveness, and perceived safety, resulting in relatively controlled spending patterns compared to chronic conditions that require ongoing pharmacotherapy (16, 17).

Preventive and wellness products demonstrated a unique bimodal distribution, with expenditures concentrated in both the 50–99 AED and 100–499 AED ranges. This suggests two distinct consumer groups: one adopting relatively low-cost supplementation practices and another investing more heavily in premium or multiple wellness products such as vitamin E or D. Similar patterns of variability in spending on supplements and herbal products have been observed in other community-based studies (9, 11, 12).

This study highlights that satisfaction with OTC medication costs is shaped by a complex interaction of demographic, socioeconomic, and clinical factors. Males, employed individuals, and those with larger households reported greater dissatisfaction, likely reflecting higher financial pressures and expectations of affordability. Income was a major determinant, with higher earners more satisfied and those without income most dissatisfied, while students appeared relatively more content, possibly due to lower health-related expenditures or external support. However, another study reported that patients younger than 35 years were more concerned about medical expenditures, whereas those aged 36 to 50 years prioritized concerns related to the quality of medical services, suggesting that perceptions of healthcare costs and satisfaction may also vary by age group (1, 2).

Daily use of multiple medications, and greater reliance on OTC products were strongly linked to dissatisfaction, emphasizing the cumulative burden of polypharmacy. Insurance coverage was a critical determinant, as participants with OTC coverage reported significantly higher satisfaction, while those facing greater out-of-pocket spending demonstrated marked dissatisfaction. A similar pattern was observed Fu and Wang (18) who reported that patients with lower insurance reimbursement ratios were more likely to express dissatisfaction with their medical expenses, reinforcing the importance of adequate financial protection in shaping perceptions of affordability.

Although adverse drug reactions did not reach statistical significance, a trend toward dissatisfaction suggests that negative medication experiences may further reduce perceived value. Collectively, these findings underscore the need for targeted interventions such as broader insurance coverage, cost-reduction policies, and support for high-risk groups to improve medication affordability and satisfaction. A similar observation was reported in other community-based studies, whereas a contradictory finding was observed in a United States study where no causal relationship could be inferred between patient satisfaction and healthcare expenditure (18).

### Limitations and future research

This study has several limitations that should be considered when interpreting the findings. First, cross-sectional design limits the ability to establish causal relationships between demographic and behavioral factors and OOP expenditure on OTC medications; therefore, only associations can be explored. Longitudinal studies are needed to better understand changes in OTC spending patterns over time.

Second, although data were collected across multiple community-based settings in the UAE to enhance participant diversity, the use of convenience sampling may limit the generalizability of the findings to the wider population. In addition, individuals without internet access, particularly older adults and those residing in rural or underserved areas, may have been underrepresented among participants who completed the digital version of the questionnaire.

Third, the relatively modest sample size limited statistical power for detecting small effect sizes and precluded robust multivariable adjusted analyses. As a result, observed associations should be interpreted with caution and considered hypothesis-generating rather than definitive.

Fourth, OOP expenditure data were self-reported and relied on participants’ recall of spending over the previous 30 days, which may be subject to recall bias or reporting inaccuracies. Furthermore, information on the specific place of purchase, such as community pharmacy, retail outlet, or online platform, and differentiation between brand-name and generic OTC medications was not systematically captured, which may have influenced the estimation of OOP expenditure.

In addition to the current findings, future research could adopt qualitative methods to gain deeper insights into consumer motivations, decision-making processes, and perceptions of risk related to OTC medication use. Longitudinal studies would be valuable for tracking evolving self-medication patterns and evaluating the sustained impact of public health strategies aimed at promoting safe practices. Further work should also explore the role of pharmacists and other healthcare professionals in guiding responsible self-medication, as enhancing consumer awareness of safety, affordability, and appropriate use could substantially improve the effective utilization of OTC products. Another important avenue for future research is the evaluation of cost-effectiveness and insurance coverage impacts, which would help to better understand the policy implications of self-medication and out-of-pocket expenditure.

## Conclusion

This study demonstrates that self-medication with over-the-counter (OTC) medications is prevalent among adults in the UAE, with females and employed individuals reporting higher usage. Analgesics, antipyretics, and respiratory treatments were the most used products, reflecting common health concerns in the population. Overall satisfaction with OTC medication costs was high, yet a notable proportion of participants incurred moderate out-of-pocket (OOP) expenses, particularly for acute illnesses. Promoting cost-effective self-medication practices, providing patient education on appropriate OTC use, and integrating considerations of financial burden into healthcare planning may help optimize both health outcomes and patient satisfaction.

## Data Availability

The datasets presented in this study can be found in online repositories. The names of the repository/repositories and accession number(s) can be found in the article/Supplementary material.
