# Supplementary material for: Out-of-pocket expenditures on over-the-counter medications: a cross-sectional study of consumer spending patterns
Source: Front Public Health. 2026 Mar 27;14:1777770. doi: 10.3389/fpubh.2026.1777770 (PMC13066303; doi:10.3389/fpubh.2026.1777770)
Supplement: Supplementary file 1 [file Table_1.docx]

**Article title:**

Out-of-Pocket Expenditures on Over-the-Counter Medications: A Cross-Sectional Study of Consumer Spending Patterns.

**Authors:**Hadel Fuad Alzuabi¹, Vineetha Bharathan Menon^2^, Jisha Myalil Lucca^3^

Supplementary materials:

**Data Collection Form:**

**Section 1: Demographic & Social Information**

Gender: (Single choice)

◯ Male

◯ Female

◯ Prefer not to say

Age: (Single choice)

◯ 18 - 29 Years

◯ 30 - 49 Years

◯ 50 - 59 Years

◯ ≥ 60 years

Nationality: (Single choice)

◯ Emirati

◯ South Asia (India, Pakistan, Bangladesh, etc.)

◯ Other Arabs

◯ Philippines

Other

Number of household members: (Single choice)

◯ Only one person

◯ Two to three people

◯ Four to eight people

◯ More than eight

Employment status: (Single choice; includes Other)

◯ Employed

◯ Unemployed

◯ Student

◯ Self-employed

◯ Retired

Other: (text)

Monthly salary: (Single choice)

◯ Less than 5,000 AED

◯ 5,000 to 10,000 AED

◯ More than 10,000 AED

◯ Prefer not to disclose

◯ I currently have no monthly salary

**Section 2 — Medical Conditions**

Do you suffer from any of the following? (You may select more than one)

☐ Hypertension (high blood pressure)

☐ Other heart disorders (myocardial infarction, arrhythmia)

☐ Other non-cardiac disorders (diabetes, asthma, bleeding problems)

☐ Other acute illnesses (infections, colds, cough) during the last 6 months

☐ I do not have any diseases

☐ Other: __________

**Section 3 — Medication use**

How many medications do you take per day when ill (acute or chronic)?

◯ I do not take any medication

◯ 1 only

◯ 2 to 4 medications

◯ More than 5 medications

How many prescribed medications (recommended by a doctor) do you usually take?

◯ I do not take any medication

◯ 1 only

◯ 2 to 4 medications

◯ More than 5 medications

How many herbal or over-the-counter (OTC) medicines do you usually take?

(Definition: Herbal and OTC medicines include vitamins, analgesics, antihistamines, etc.)

◯ I do not take any medication

◯ 1 only

◯ 2 to 4 medications

◯ More than 5 medications

For what purpose(s) do you take OTC/herbal medicines? (Select all that apply)

☐ To treat an existing condition

☐ To help control my other diseases

☐ To prevent getting diseases

☐ For my general health

☐ Other: __________

Does your medical insurance cover the cost of OTC/herbal medicines?

◯ Yes

◯ No

◯ I don’t know

◯ I don’t have health insurance

What is the monthly out-of-pocket cost you pay to buy herbal/OTC medicines?

◯ 0 (zero)

◯ Less than 50 AED

◯ 50 to 100 AED

◯ 100 to 500 AED

◯ 500 to 1000 AED

◯ More than 1000 AED

How much do you spend on herbal/OTC products for the following conditions?

| **Condition \ Cost** | **0 (zero)** | **<50 AED** | **50–100 AED** | **100–500 AED** | **500–1000 AED** | **>1000 AED** |
| --- | --- | --- | --- | --- | --- | --- |
| Hypertension | ◯ | ◯ | ◯ | ◯ | ◯ | ◯ |
| Other heart disorders (MI, arrhythmia) | ◯ | ◯ | ◯ | ◯ | ◯ | ◯ |
| Other non-cardiac disorders (diabetes, asthma) | ◯ | ◯ | ◯ | ◯ | ◯ | ◯ |
| Other acute illnesses (infections, colds, cough) | ◯ | ◯ | ◯ | ◯ | ◯ | ◯ |
| Other (vitamins, minerals, skincare) | ◯ | ◯ | ◯ | ◯ | ◯ | ◯ |

Who recommended you these herbal/OTC products?

◯ Health care professionals

◯ Friends and relatives

◯ Advertising/marketing

◯ Other: __________

Have you ever experienced any side effects due to herbal or OTC medicines?

◯ Yes

◯ No

How much do you adhere to label or pharmacist recommendations regarding herbal/OTC medicines? (adherence scale)

◯ I never adhere

◯ 1

◯ 2

◯ 3

◯ 4

◯ 5

◯ I adhere completely

**Section 4 — Reasons for use & product types**

Why do you prefer to buy OTC or herbal medicines? (Select all that apply)

☐ I do not like visiting the hospital

☐ If the illness is mild, I can treat it myself

☐ I keep them at home for emergencies

☐ It is traditionally used by my family/friends

☐ Because it is cheaper than other products

☐ I think it is safer than other medicines

☐ I think it is more effective than other medicines

☐ Other: __________

Which herbal/OTC products do you usually use?

| **Product \ Frequency** | **Never** | **Sometimes** | **Often** | **Always** |
| --- | --- | --- | --- | --- |
| Analgesics (painkillers) | ◯ | ◯ | ◯ | ◯ |
| Cough & cold medicines | ◯ | ◯ | ◯ | ◯ |
| Vitamins & minerals | ◯ | ◯ | ◯ | ◯ |
| Sexual enhancement products | ◯ | ◯ | ◯ | ◯ |
| Stomach problems (indigestion, diarrhea) | ◯ | ◯ | ◯ | ◯ |
| Herbal products | ◯ | ◯ | ◯ | ◯ |
| Mild sleeping aids | ◯ | ◯ | ◯ | ◯ |

**Section 5 — Satisfaction**

Overall, what is your level of satisfaction with the following regarding herbal/OTC products?

| **Item \ Satisfaction** | **Extremely satisfied** | **Satisfied** | **Neutral** | **Dissatisfied** | **Extremely dissatisfied** |
| --- | --- | --- | --- | --- | --- |
| Cost of OTC products | ◯ | ◯ | ◯ | ◯ | ◯ |
| Effectiveness of OTC products | ◯ | ◯ | ◯ | ◯ | ◯ |
| Safety of OTC products (side effects) | ◯ | ◯ | ◯ | ◯ | ◯ |
| Availability of OTC products | ◯ | ◯ | ◯ | ◯ | ◯ |

Do you think insurance companies should cover OTC and herbal products?

◯ Yes

◯ No

◯ Maybe
